# *Meloidogyne enterolobii*-induced Changes in Guava Root Exudates Are Associated With Root Rotting Caused by *Neocosmospora falciformis*

**DOI:** 10.2478/jofnem-2023-0055

**Published:** 2023-12-31

**Authors:** Ricardo M. Souza, Denilson F. Oliveira, Vicente M. Gomes, Abraão J. S. Viana, Geraldo H. Silva, Alan R. T. Machado

**Affiliations:** Departamento de Entomologia e Fitopatologia, Universidade Estadual do Norte Fluminense Darcy Ribeiro, Campos dos Goytacazes, Brazil; Departamento de Química, Universidade Federal de Lavras, Lavras, Brazil; Laboratório de Desenvolvimento de Agroquímicos Naturais, Universidade Federal de Viçosa, Rio Paranaíba, Brazil; Departamento de Ciências Exatas, Universidade do Estado de Minas Gerais, João Monlevade, Brazil

**Keywords:** 1,5-dinitrobiuret, disease complex, guava, guava decline, *Meloidogyne enterolobii*, nematode-fungus interaction, *Neocosmospora falciformis*, *Psidium guajava*, root exudate, root-knot nematode

## Abstract

Despite the worldwide importance of disease complexes involving root-feeding nematodes and soilborne fungi, there have been few in-depth studies on how these organisms interact at the molecular level. Previous studies of guava decline have shown that root exudates from *Meloidogyne enterolobii*-parasitized guava plants (NP plants), but not from nematode-free plants (NF plants), enable the fungus *Neocosmospora falciformis* to rot guava roots, leading to plant death. To further characterize this interaction, NP and NF root exudates were lyophilized; extracted with distinct solvents; quantified regarding amino acids, soluble carbohydrates, sucrose, phenols, and alkaloids; and submitted to a bioassay to determine their ability to enable *N. falciformis* to rot the guava seedlings’ roots. NP root exudates were richer than NF root exudates in amino acids, carbohydrates, and sucrose. Only the fractions NP-03 and NP-04 enabled fungal root rotting. NP-03 was then sequentially fractionated through chromatographic silica columns. At each step, the main fractions were reassessed in bioassay. The final fraction that enabled fungal root rotting was submitted to analysis using high performance liquid chromatography, nuclear magnetic resonance, mass spectrometry, energy-dispersive X-ray fluorescence, and computational calculations, leading to the identification of 1,5-dinitrobiuret as the predominant substance. In conclusion, parasitism by *M. enterolobii* causes an enrichment of guava root exudates that likely favors microorganisms capable of producing 1,5-dinitrobiuret in the rhizosphere. The accumulation of biuret, a known phytotoxic substance, possibly hampers root physiology and the innate immunity of guava to *N. falciformis*.

Guava decline is the most important phytosanitary problem in the guava (*Psidium guajava* L.) crop in Brazil. First reported in Brazil in 1989 with an erroneous etiology, by 2009 it had spread to over 5,000 hectares and caused over US $60 million in losses (Pereira et al., 2009). In this disease complex, parasitism by *Meloidogyne enterolobii* Yang and Eisenback predisposes the plant to extensive root rotting caused by *Neocosmospora falciformis* (Carrión) L. Lombard and Crous, a fungus to which guava is immune (i.e., the fungus cannot infect and damage the plant) (Gomes et al., 2011; Veloso et al., 2021). Above ground, foliar deficiency in nitrogen, phosphorus and potassium follows, along with an accumulation of manganese, chlorine, and sodium to near-phytotoxic levels. These are accompanied by leaf chlorosis, scorching of margins and wilting, and total defoliation of the tree (Gomes et al., 2008). Typically, the plant dies a few months after onset of symptoms.

Disease complexes - also referred to as synergistic interactions - involving phytonematodes and soilborne fungi have been reported in several crops (Evans and Haydock, 1993; Francl and Wheeler, 1993; Abawi and Chen, 1998; Back et al., 2002). Intractable as these pathogens are on their own, disease complexes pose additional hurdles to crop management. In recent years, disease complexes have caused major losses in soybean in the US, coffee in Central America, guava in Brazil, and cotton worldwide (Pereira et al., 2009; Villain et al., 2013; Sanogo and Zhang, 2015; Navi and Yang, 2016).

Despite the importance of disease complexes, researchers still have a narrow understanding of how nematodes, soilborne fungi and plants interact at the genetic, molecular and physiological levels (Abawi and Chen, 1998; Manzanilla-López and Starr, 2009). For instance, the rupture of root epidermis and cortex by the nematode, creating entry points for the fungus, seems important in some synergistic interactions such as between *Pratylenchus penetrans* (Cobb) Filipjev and Schuurmans Stekhoven and *Verticillium albo-atrum* Reinke and Berthold, as well as the one between *Meloidogyne incognita* (Kofoid and White) Chitwood and *Rhizoctonia solani* Kühn, both of which occur in tomato (Conroy et al., 1972; Golden and Van Gundy, 1975). Nematode and fungal impacts on physiological processes such as the uptake and transport of water and nutrients also contribute to synergistic losses (Hajihassani et al., 2013).

In other synergistic interactions, nematode parasitism seems to hamper plant resistance to fungi. For example, parasitism by *M. incognita* and *M. javanica* (Treub) Chitwood hampers pigeon pea resistance to *Fusarium udum* Butler due to reduced synthesis of the phytoalexin cajanol (Marley and Hillocks, 1994). Conversely, no mechanism has been proposed for interactions in which the fungus renders the plant susceptible to nematodes (Hasan, 1985; Hasan and Khan, 1985; Khan and Husain, 1989; Taheri et al., 1994; Zahid et al., 2002).

A third arena for synergistic interactions is root exudates. Root exudates refer to a suite of substances—mostly organic acids, sugars, and amino acids—that are secreted by plant roots (Koo et al., 2005). The amount and composition of root exudates respond to phenology and (a)biotic factors that interfere with the plant’s physiology (Vives-Peris et al., 2020). The rhizobiome, composed of free-living microorganisms as well as those that are symbiotic with plants or pathogenic to them, responds to and modifies root exudates (Koo et al 2005; Bashir et al. 2016; Sanchez-Cañizares et al. 2017; Sasse et al., 2018).

Evidence is mounting that root exudates and the rhizobiome are critical to plant health (Berendsen et al., 2012). In this context, the incidence and severity of a soilborne disease is not determined by the pathogen alone, but rather as a result of interactions between the plant and its rhizobiome, mediated by root exudates. Conversely, the onset of a soilborne disease affects the composition of root exudates, which impacts the rhizobiome. For instance, tomato wilt caused by *Raustonia solanacearum* (Smith) Yabuuchi, Kosako, Yano, Hotta, and Nishiuchi changed the chemical profile of root exudates and altered the rhizosphere’s bacterial community (Gu et al., 2016; Wey et al., 2018). Lisianthus wilt caused by *F. oxysporum* Schlecht changed the root exudate composition as well as the rhizosphere’s bacterial and fungal communities (Huang et al., 2019, 2020).

Root-feeding nematodes also induce changes in root exudates, which impact the rhizobiome. Parasitism by *M. incognita* led to an increase of sugars and electrolytes in tomato root exudates (Wang and Bergeson, 1974; Wang et al., 1975). Several other nematodes have also been found to cause “leaking” of carbon- and nitrogen-rich compounds, resulting in changes in the rhizosphere’s microbial community (Yeates et al., 1998, 1999; Denton et al. 1999; Tu et al., 2003; Dromph et al., 2006; Hasse et al., 2007; Wurst et al. 2009; Tian et al., 2015; Lamelas et al., 2020; Xiaolong et al., 2022).

Khan (1993) and Evans and Haydock (1993) suggested that changes in root exudates could be involved in synergistic interactions involving nematodes and soilborne fungi. This has been investigated for only two disease complexes. In tomato, *M. incognita* parasitism caused root exudates to become enriched with electrolytes, sugars, and nitrogen-rich compounds, which favored root decay caused by *R. solani* (van Gundy et al. 1977). In guava decline, root exudates collected from plants parasitized by the nematode *M. enterolobii* (nematode-parasitized plants, NP), but not from nematode-free plants (NF plants), had a positive effect on mycelial growth and propagule production of *N. falciformis* (Gomes et al., 2013). This suggested a nutritional and/or physiological effect of NP root exudates on the fungus. However, this putative effect occurred only when the nematode and the fungus were co-inoculated in split-root assays. When they were inoculated in distinct root systems, no root rot occurred (Gomes et al., 2014).

The role of NP root exudates in enabling *N. falciformis* to cause decay in guava roots was confirmed in pathogenicity tests. In guava seedlings, *N. falciformis* reduced shoot and root fresh masses and caused root decay and damping-off, but only in the presence of NP root exudates (Gomes et al., 2013). Both unlyophilized and lyophilized and resuspended NP root exudates enabled *N. falciformis*’s pathogenicity, which indicated that the chemical substance(s) involved is neither volatile nor proteic in nature.

This study aimed to further characterize the substance(s) present in NP root exudates that enable *N. falciformis* to cause decay in guava roots. To accomplish this, NP and NF root exudates were lyophilized; extracted with distinct solvents; quantified for amino acids, soluble carbohydrates, sucrose, phenols, and alkaloids components; and sequentially fractionated through chromatographic silica columns. At each step, the main fractions were assessed through bioassays for their ability to enable *N. falciformis* to decay guava roots. The final fungus-enabling fractions were submitted to analysis by high performance liquid chromatography (HPLC), nuclear magnetic resonance (NMR), mass spectrometry, energy-dispersive X-ray fluorescence (EDXRF), and computational calculations. This study brings an unprecedented characterization of root exudate components involved in synergistic interactions involving *Meloidogyne* spp. and soilborne fungi.

## Material and Methods

### Seedling cultivation and inoculation

The procedures followed those outlined by Gomes et al. (2013). Briefly, seedlings of *M. enterolobii*-susceptible guava ‘Paluma’ were produced from stem cuttings and individually transferred to black plastic plant-growth tubes filled with 100 mL of autoclaved, washed riverbed sand. The base of each tube was closed with a plug of sterile cotton wool. The plants were kept in a growth chamber under a 12-hr photoperiod, with illumination of 35 μmol/(m^2^/s) provided by Grow-Lux (Sylvania, Lisboa, Portugal) light tubes. The day/night temperatures were set at 25 and 23 °C, respectively. The plants were watered with sterile, distilled water as needed. Biweekly, the plants were sprayed with foliar fertilizer Ouro Verde (Mato Verde Jardinagem, Bady Bassitt, Brazil), which provided nitrogen (urea), phosphorus (H_3_PO_4_), potassium (KCl), manganese, iron, boron, zinc, calcium, sulfur, and magnesium. For fertilizer application, the sand was covered with a plastic sheet to avoid getting drops on it.

*Meloidogyne enterolobii* was collected from a guava orchard (location coordinates: −21.649131; −41.042880) affected by guava decline. The nematode identity was ascertained by morphology and esterase phenotyping (Esbenshade and Triantaphyllou, 1985). The nematode was cultured in greenhouse-grown tomato plants. For inoculum preparation, tomato roots were cleaned with tap water and processed for nematode extraction through a modified version of the method detailed by Coolen and D’Herde (1972), i.e., without adding kaolin in the blender. The resulting suspension was passed through 60- and 500-mesh sieves (250- and 25-μm openings, respectively). The nematode eggs and second-stage juveniles (J_2_) retained in the 500-mesh sieve were resuspended in water, and three 1-mL aliquots were counted on Peter’s slides under a stereomicroscope. The inoculum was calibrated to 200 eggs + J_2_/mL of water. When the plants had rooted well and emitted two pairs of true leaves, 10 mL of inoculum (2000 eggs + J_2_) were applied per plant, in two 3-cm-deep holes that were made in the sand around the plant. One hundred plants were inoculated (NP plants) and 100 plants received water only (NF plants).

### Collection of root exudates

When NP plants had developed abundant root galls, watering was suspended for 72 hr. The NP and NF plants were then watered with 50 mL of sterile, distilled water. This resulted in about 10 mL of root exudate per plant, which percolated through the cotton-wool plug. The NP and NF root exudates were separately pooled in sterile glass flasks wrapped in aluminum foil to minimize exposure to light. The root exudates were passed twice through a Whatman #1 paper filter (Whatman plc, Maidstone, UK) using a vacuum pump, and through a 0.22-μm-opening Teflon (Chemours, Wilmington, DE, USA) Millipore (MilliporeSigma, Burlington, MA, USA) filter membrane using the TTP vacuum filtration system (TTP, Switzerland). The exudates were collected into sterile, sealed TTP containers, frozen immediately, and then lyophilized in an L101 lyophilizer (Liotop, Brazil) within 2–3 days. Over about eight months, the 100 NP and 100 NF plants provided about 40 L of root exudates in each group. After lyophilization, this resulted in 23.1 g of NP and 16.4 g of NF dried matter.

### Solvent extraction of NP and NF dried matter

Extracts were obtained with organic solvents of increasing polarity - hexane, ethyl acetate, methanol, and water. The NP dry matter (21.1 g) was immersed in 150 mL of hexane and agitated for 30 min at room temperature. The suspension was filtered through a cotton wool plug, and the solid residue underwent two more extractions with 150 mL of hexane each. The three liquid fractions were pooled, concentrated to dryness in a rotary evaporator, and lyophilized. The hexane-insoluble residue was then submitted to sequential extractions with ethyl acetate (3 × 150 mL), methanol (3 × 150 mL) and water (3 × 150 mL). The same extraction procedure was followed with the 14.6 g of NF dried matter, except that the volume of the solvents was 100 mL. The masses of the hexane, ethyl acetate, methanol, and water NP extracts (NP-01, NP-02, NP-03, and NP-04, respectively), and of the NF extracts (NF-01, NF-02, NF-03, and NF-04) are shown in Supplementary Table 1.

### Bioassay 1 – effect of NP and NF extracts on the pathogenicity of *N. alciformis* to guava seedlings

**Figure 1:**
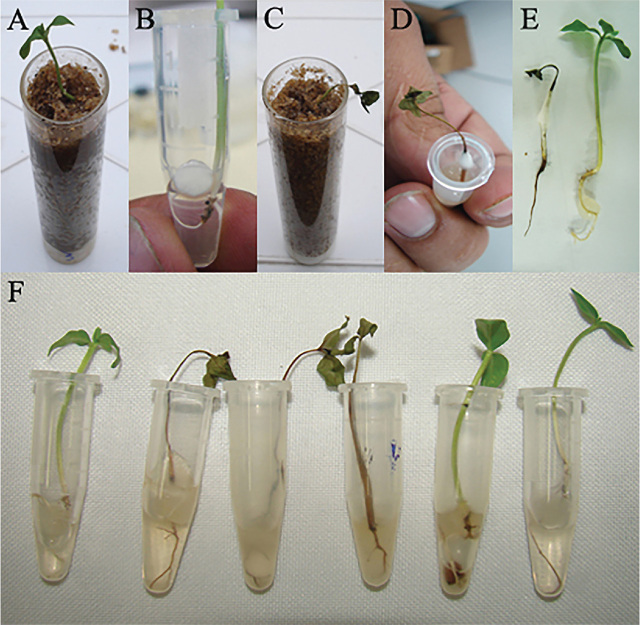
Effect of extracts and fractions of guava root exudates on the pathogenicity of *Neocosmospora falciformis* to guava seedlings. Root exudates (coded NP) were collected from plants cultivated in a growth chamber and parasitized by *Meloidogyne enterolobii*. A) Uninoculated seedling in glass tube filled with autoclaved sand (bioassay 1). B) Seedling in an Eppendorf tube, inoculated with a fungus-colonized agar-water plug positioned in the collar region (bioassay 2). C) Damping-off in a seedling inoculated with the fungus and watered with an aqueous solution prepared from the extract NP-03. D) Damping-off and fungal growth in a seedling watered with an aqueous solution prepared from the fraction NP-03-F2. E) Fungus-inoculated seedlings watered with an aqueous solution prepared from NP-03-F2 (left) or sterile, distilled water (right). F) From left to right: fungus-inoculated seedlings watered with sterile, distilled water or fractions NP-03-F2, NP-03-F2-3, NP-03-F2-4, NP-03-F2-5 or NP-03-F2-6 (bioassay 3).

Seeds of guava ‘Paluma’ were germinated in autoclaved sand. When the cotyledon leaves had spread, the seedlings were sequentially rinsed with sterile, distilled water, immersed in commercial bleach at 1% active chlorine for 1 min, and then rinsed with sterile, distilled water. The seedlings were then individually transferred to sterile 30-mL glass tubes filled with 20 g of autoclaved, washed riverbed sand (Fig. 1A). The seedlings were maintained in a growth chamber under a 12-h photoperiod, with an illumination of 35 μmol/(m^2^/s^1^) provided by Grow-Lux light tubes. The day and night temperatures were set at 25 and 23 °C, respectively.

*Neocosmospora falciformis* isolate UENF/CF 163 originated from an orchard (location coordinates: −21.649131; −41.042880) affected by guava decline (Gomes et al., 2011). This isolate was maintained in anhydrous silica gel. To keep it from losing its pathogenicity to guava, it was routinely revived in potato-dextrose-agar medium, inoculated in guava seedlings, re-isolated, and preserved.

For seedling inoculation, *N. falciformis* was cultured in 1.5%-agar-water medium. A 0.5-cm plug was collected from the edge of the colony and positioned in the seedling collar region. The inoculated seedlings were watered daily with 2-mL aqueous solutions prepared from the NP-01, NP-02, NP-03, NP-04, NF-01, NF-02, NF-03 or NF-04 extracts. These watering solutions were prepared so as to match the extract concentration in the root exudates collected from the guava plants, according to the formula C_F_ = (C_E_ × m_F_)/m_E_, in which C_F_ is the fraction (NP-01 to NP-04 or NF-01 to NF-04) concentration in the watering solution; C_E_ is the concentration of the exudate solution; m_F_ is the fraction mass; and m_E_ is the exudate mass from which the fraction was obtained. The blank controls consisted of fungus-inoculated and uninoculated seedlings watered with 2 mL of sterile, distilled water. Each of the 10 treatments had eight replicates, for a total of 80 seedlings. The seedlings were arranged in trays in the growth chamber in a completely randomized design, separated from each other by a few centimeters.

Fifteen days after fungus inoculation, the seedlings were carefully removed from the glass tubes and examined for collar and rootlet necrosis. In the treatments where the fungus caused necrosis (see Results), the tissues decayed quickly, so we measured the rootlets’ unrotten fresh weight (mg) and the shoots’ fresh weight (mg). This bioassay was repeated once.

Data from both bioassays were tested for homogeneity of variances (Cochran and Bartlett tests) and for normality of errors (Lilliefors test), at 5% probability. No data transformation was necessary. Data were submitted to ANOVA, with time being one of the factors. Since the *F*-test revealed no significance (*p* > 0.05) for the factor time, data from both assays were pooled. The treatment means were compared by the Tukey HSD test at 5% probability.

### Quantification of free amino acids, soluble carbohydrates, sucrose, phenols and alkaloids in the methanol and water NP and NF extracts

Only NP-03 and NP-04 enabled *N. falcifomis* pathogenicity to guava seedlings (see Results). Quantification of amino acids, soluble carbohydrates, and sucrose (mg/mL) in NP-03, NP-04, NF-03 and NF-04 were performed using the method developed by Passos (1996) and modified by Campos et al. (2012). Phenol quantification (mg/mL) was performed using a modified version of the method of the Association of Official Analytical Chemists (Anonymous, 1996), and alkaloids were quantified (mg/mL) using of Sreevidya and Mehrotra (2003)’s method. A detailed description of these methods is contained in the Supplementary Material and Methods. All quantifications were conducted twice.

For each chemical group or substance, data were tested for homogeneity of variances (Cochran and Bartlett tests) and for normality of errors (Lilliefors test), at 5% probability. No data transformation was necessary. Data were submitted to ANOVA, with time being one of the factors. Since the *F*-test revealed no significance (*p* > 0.05) for the factor time, data from both quantifications were pooled. For each chemical group/substance, the concentrations in the different extracts were compared by the Tukey test at 5% probability.

### Fractionation of NP-03

NP-03 and NP-04 were the extracts that enabled *N. falciformis* pathogenicity, and the former had considerably more mass (Supplementary Table 1), and thus it was chosen for further characterizations. Adapting the method of Still et al. (1978), a NP-03 aliquot of 4.6 g underwent fractionation by elution through a 7 × 15-cm column of flash silica gel (Merck, 40-63 μm). Five eluents were consecutively used (700 mL each): ethyl acetate/methanol (9:1, v/v); methanol; water; water/acetic acid (99:1, v/v); and 0.1 mol/L aqueous hydrochloric acid. The five fractions obtained were concentrated to dryness in a rotary evaporator, and lyophilized. Their masses were 0.017 g (NP-03-F1), 3.782 g (NP-03-F2), 0.135 g (NP-03-F3), 0.254 g (NP-03-F4), and 0.266 g (NP-03-F5).

### Bioassay 2: effect of NP-03 fractions on the pathogenicity of *N. falciformis* to guava seedlings

The seedlings were germinated, inoculated, and watered like in bioassay 1, with one adaptation: the guava seedlings were maintained in water in Eppendorf tubes (Figure 1B) (Eppendorf, Hamburg, Germany). This reduced the volume of the watering solution to 1 mL daily. Consequently, less of the fractions' mass was spent to prepare the solutions. To prepare the watering solutions from the NP-03 fractions, we applied the formula described before. The blank controls consisted of fungus-inoculated and uninoculated seedlings watered with 2 mL of sterile, distilled water. Eight replicates were used for each treatment, for a total of 56 seedlings. Evaluations were conducted as described before. This bioassay was repeated once, and the statistical analysis was conducted as previously described.

### Chromatographic fractionation of NP-03-F2

Only NP-03-F2 enabled *N. falcifomis* pathogenicity to guava seedlings (see Results). NP-03-F2 was analyzed by thin layer chromatography (TLC), using silica-coated aluminum plates with a 250-μm-thick layer of fluorescent indicator (Merck, Germany). Initially, various solvents and combinations thereof were used for vertical development. For visualization of the substances after development, the plates were dried (~70 °C) and observed under both daylight and 254-nm UV light, before and after derivatization, with either iodine vapor or ninhydrin at 0.002g/mL in ethanol. The best result was obtained using ethyl acetate/methanol (7:3) for development, which afforded a retention factor (Still et al., 1978) of around 0.23 for the major components.

A 1.52-g aliquot of NP-03-F2 was eluted through a 4-by-15-cm flash silica gel column, with ethyl acetate/methanol (7:3; 600 mL), ethyl acetate/methanol (3:7, 400 mL), and water (400 mL). Twenty-nine 40-mL fractions were collected, except for the eluent water, for which a single fraction was collected. Fractions that were similar according to TLC analysis were pooled, resulting in eight fractions that were coded NP-03-F2-1 through NP-03-F2-8. These fractions were concentrated to dryness in a rotary evaporator and lyophilized. Their masses are shown in Supplementary Table 2.

### Bioassay 3 – effect of NP-03-F2 fractions on the pathogenicity of *N. falciformis* to guava seedlings

Fractions NP-03-F2-3, NP-03-F2-4, NP-03-F2-5 and NP-03-F2-6, which had the greatest masses, underwent this bioassay. The procedures for seedling germination, inoculation, preparation of the watering solutions, and watering were as in bioassay 2. The controls consisted of fungus-inoculated seedlings, watered with sterile, distilled water or fraction NP-03-F2. Eight replicates were used for each treatment, for a total of 48 seedlings. Evaluations were conducted as described before, but a less-severe decay of tissues allowed us also to measure the rootlets’ rotten fresh mass (mg). This bioassay was repeated once. The statistical analysis was conducted as described before.

### Fractionation of NP-03-F2-3 and NP-03-F2-4

Only NP-03-F2-3 and NP-03-F2-4 enabled *N. falciformis* pathogenicity in guava seedlings (see Results). These fractions were combined due to their similar chromatographic behavior and were thereafter referred to as NP-03-F2-3+4. A 371.8-mg aliquot of NP-03-F2-3+4 underwent consecutive extractions with diethyl ether (2 × 35 mL), ethanol (4 × 35 mL), and methanol (4 × 35 mL). The liquid fractions and the final residue were concentrated to dryness in a rotary evaporator and lyophilized. Their masses are in the Supplementary Table 3.

The fraction with the greatest mass—NP-03-F2-3+4-ET—was eluted through a flash silica gel column with ethyl acetate/methanol (8:2, 400 mL; 6:4, 400 mL; 1:1, 200 mL). Fifty 20-mL fractions were collected, followed by a single 300-mL methanol fraction. The fractions were analyzed by TLC using silica-coated aluminum plates impregnated with fluorescent indicator F254. For visualization of the substances after development, the plates were dried (~70 °C) and observed under daylight and 254-nm UV light, before and after exposure to iodine vapor. The plates were then dipped in a solution of 0.07 g/mL of phosphomolybdic acid in ethanol, and heated. The fractions were pooled according to the TLC analysis, concentrated to dryness in a rotary evaporator, and lyophilized. Their masses are enumerated in Supplementary Table 4.

### Analyses of the substances present in the fractions NP-03-F2-3+4-ET-02 and NP-03-F2-3+4-ET-05

Fractions NP-03-F2-3+4-ET-02 and NP-03-F2-3+4-ET-05 had pure substances according to TLC analysis. Therefore, they were analyzed by HPLC in a Shimadzu (Kyoto, Japan) apparatus equipped with a DAD SPD-M20A-type UV-visible detector, two pumps LC-6AD, Rheodyne 7725i-type manual injector (all also Shimadzu, Kyoto, Japan), and a Luna 5μm column (phenyl-hexyl, 250 by 4.60 mm) (Phenomenex, Torrance, CA, USA). The eluent was ultrapure water. The fractions were also submitted to elemental analysis in an Truspec Micro Element Analyzer (LECO, USA). Approximately 2 mg of each fraction was incinerated at 1075 °C in a quartz tube, and the resulting gases were analyzed with infrared and thermal detectors for carbon and nitrogen quantification, respectively.

The fractions were also submitted to analysis by EDXRF using a Shimadzu EDX-720. This analysis was carried out in the qualitative/quantitative mode under reduced pressure, for detection of chemical elements from ^11^Na to ^238^U. To obtain ^1^H and ^13^C NMR spectra, the fractions were dissolved in hexadeuterated dimethylsulfoxide (DMSO-*d*_6_), and analyzed in an Avance III (Bruker, Carteret, NJ, USA) NMR spectrometer (^1^H: 600.13 MHz; ^13^C: 150.9 MHz). To obtain the mass spectra, the fractions were dissolved in methanol, and injected into a Daltonics microTOF-Q apparatus (Bruker, Carteret, NJ, USA) equipped with an ESI-type ionizer.

### Computational calculations applied to 1,5-dinitrobiuret

**Supplementary Figure 1:**
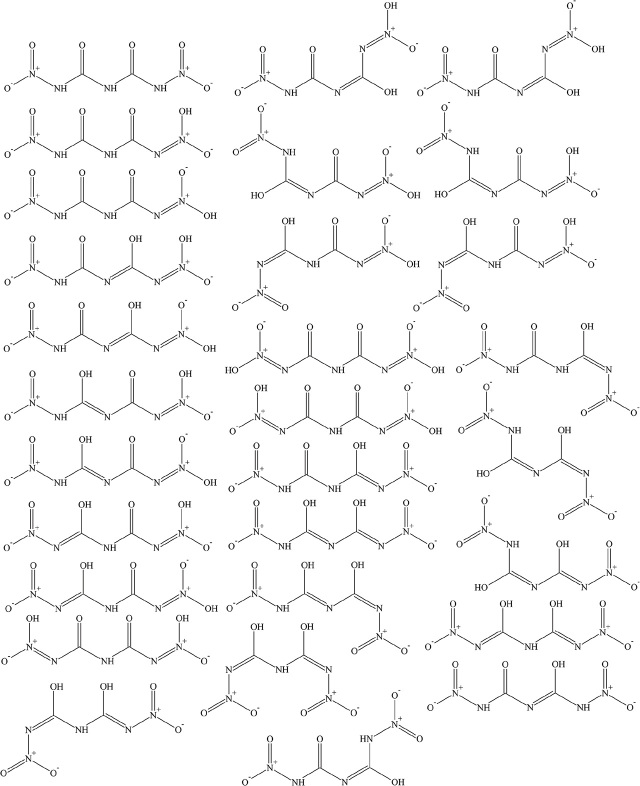
Chemical structure of 1,5-dinitrobiuret and all its tautomers, which were submitted to computational calculations with dimethylsulfoxide (DMSO) as the solvent.

Data from mass spectrometry, elemental analysis, NMR and EDXRF indicated 1,5-dinitrobiuret as the substance present in the fraction NP-03-F2-3+4-ET-05 (see Results). Therefore, conformational searches for all possible tautomers of 1,5-dinitrobiuret were undertaken, taking into account the geometric arrangements around the generated double bonds (Supplementary Fig. 1). For this, we used the computer programs Chemsketch 12.01 (Advanced Chemistry Development, Toronto, Canada); OpenBabel 2.3.2 (O’Boyle et al., 2011); Open3Dalign 2.282 (Tosco et al., 2011); Mopac 2016 (http://openmopac.net/, J.J.P. Stewart, Stewart Computational Chemistry, Colorado Springs, CO, USA), with the conductor-like screening model COSMO (Klamt and Shüürmann, 1993); and NWChem 6.8.1 (Aprà et al., 2020). A detailed description of the computational calculations is contained in the Supplementary Material and Methods.

### Interaction of 1,5-dinitrobiuret biuret hydrolase

**Supplementary Figure 2:**
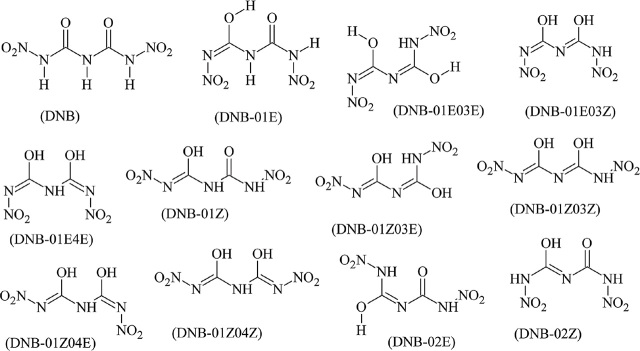
Chemical structure of 1,5-dinitrobiuret and its tautomers, which were submitted to computational calculations with water as the solvent.

**Supplementary Figure 3:**
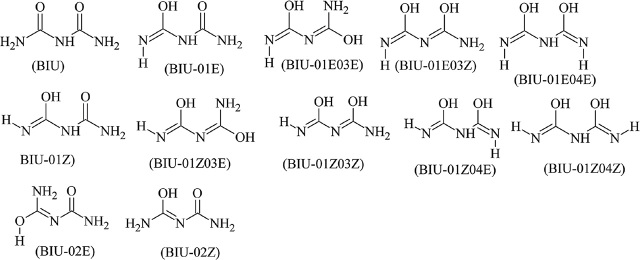
Chemical structure of biuret and its tautomers, which were submitted to computational calculations with water as the solvent.

**Supplementary Figure 4:**
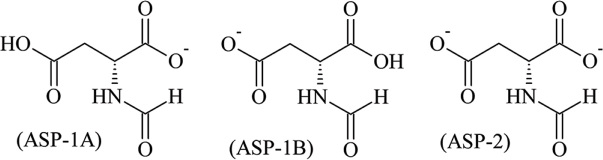
Protonation states of *N*-formyl-D-aspartic acid that were considered during computational calculations.

**Supplementary Figure 5:**
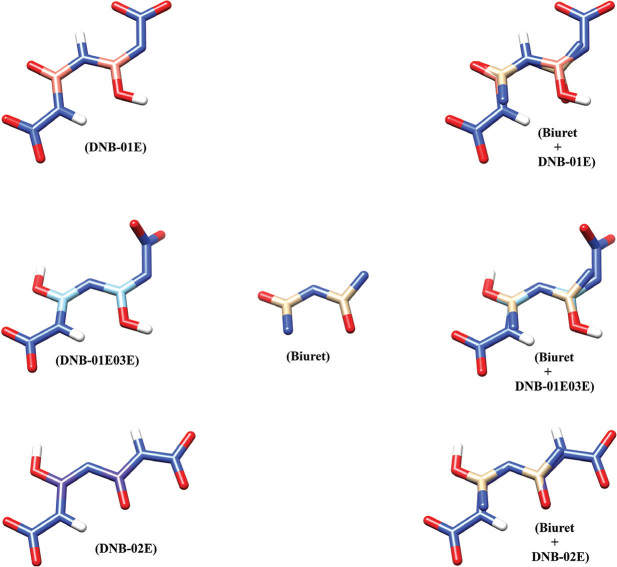
Examples of alignment of the most stable conformations of 1,5-dinitrobiuret (containing hydrogen atoms) to the structure of biuret (without hydrogen atoms), experimentally complexed to biuret hydrolase 6azq.

**Supplementary Figure 6:**
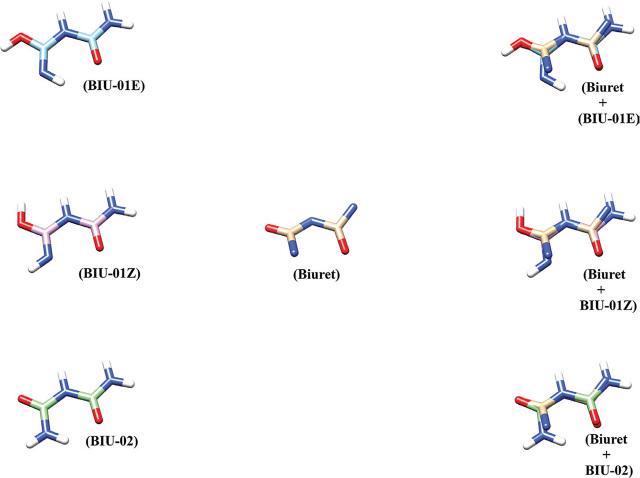
Examples of alignment of the most stable conformations of biuret (containing hydrogen atoms) to the structure of biuret (without hydrogen atoms), experimentally complexed to biuret hydrolase 6AZQ.

**Supplementary Figure 7:**
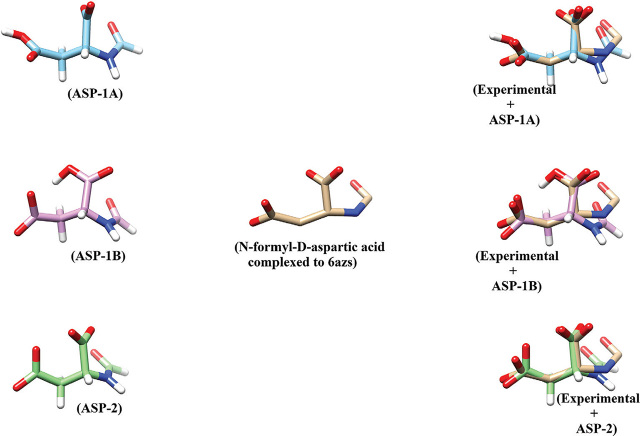
Examples of alignment of the most stable conformations of different protonation states of *N*-formyl-D-aspartic acid (containing hydrogen atoms) to the structure of *N*-formyl-D-aspartic acid (without hydrogen atoms), experimentally complexed to biuret hydrolase 6AZS.

After the conformational searches described above, 1,5-dinitrobiuret and its tautomers, biuret and its tautomers, and different protonation states of *N*-formyl-D-aspartic acid (Supplementary Figs. 2, 3, and 4, respectively), were inserted into the binding sites of biuret hydrolases (Esquirol et al., 2018) obtained from the RCSB Protein Data Bank (https://www.rcsb.org/), using the computer programs Lovoalign 21.027 (Martínez et al., 2007) and UCSF Chimera 1.15 (Pettersen et al., 2004). The resulting complexes were submitted to an optimization step with Chimera 1.15 and Antechamber (Wang et al., 2006), which employed the General Amber Force Field (GAFF) (Wang et al., 2004). Cyscore 2.0.3 (Cao and Li, 2014) was then used to calculate the affinities of the substances to the enzymes. A detailed description of these procedures is available in the Supplementary Material and Methods (Supplementary Figures 5–7). The resulting values underwent normality (Shapiro-Wilk) and homoscedasticity (Bartlett) tests at 5% probability (Snedecor and Cochran, 1989). Data was submitted to ANOVA, and means were compared using the Scott-Knott test at 5% probability (Scott and Knott, 1974), employing the R software.

## Results

### Solvent extraction of NP and NF root exudates, and effect of the extracts on *N. falciformis* pathogenicity

Virtually all masses in the NP and NF root exudates were soluble in water or methanol (Supplementary Table 1). Among all extracts, only NP-03 and NP-04 enabled *N. falciformis* pathogenicity to guava seedlings (DF = 9; F = 6.4721; *p* < 0.05) (Table 1, Fig. 1C).

**Table 1.** Bioassay 1. Effect of extracts of guava root exudates on the pathogenicity of *Neocosmospora falciformis* to guava seedlings. Root exudates were collected from plants cultivated in a growth chamber that were either parasitized by *Meloidogyne enterolobii* (coded NP) or nematode-free (NF). The solvents used were hexane (extracts -01), ethyl acetate (-02), methanol (-03) and water (-04). The extracts were used to prepare the seedlings’ watering solutions.

**Treatments**	**Rootlets’ unrotten, fresh mass**	**Fresh shoot mass**
Uninoculated seedlings watered with sterile, distilled water (control)	64.4^*^a	185.2a
Inoculated seedlings watered with sterile, distilled water (control)	64.7a	198.9a
Inoculated seedlings watered with NP-01	63.5a	198.6a
Inoculated seedlings watered with NP-02	67.1a	163.2a,b
Inoculated seedlings watered with NP-03	11.5b	84.9c
Inoculated seedlings watered with NP-04	15.9b	79.6c
Inoculated seedlings watered with NF-01	74.1a	149.7b
Inoculated seedlings watered with NF-02	66.1a	167.7a,b
Inoculated seedlings watered with NF-03	65.7a	184.2a
Inoculated seedlings watered with NF-04	67.2a	189.7a
CV%	12.1	7.7

* Values (all in mg) are the mean of two assays, each with eight replicates (seedlings) per treatment. In the columns, distinct letters indicate significant difference according to the Tukey test at 5%.

### Quantification of free amino acids, soluble carbohydrates, sucrose, phenols and alkaloids in the methanol and water NP and NF extracts

**Figure 2:**
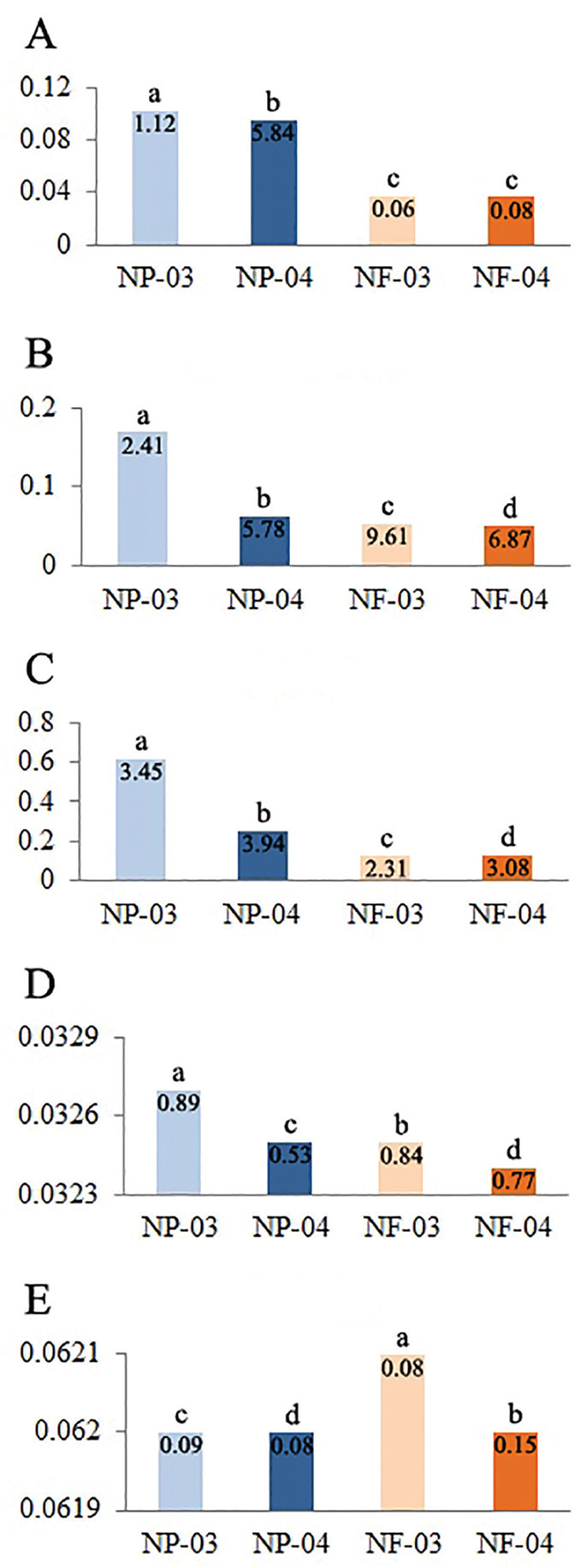
Concentration (mg/mL) of amino acids (A), carbohydrates (B), sucrose (C), phenols (D), and alkaloids (E) in extracts of guava root exudates. The guava plants were cultivated in a growth chamber, and they were parasitized by *Meloidogyne enterolobii* (coded NP) or nematode-free (NF). The solvents used to obtain the extracts were methanol (coded -03) or water (-04). Values are means of two quantifications with three replicates each. Different letters on top of the columns indicate difference according to Tukey test at 5%. In the columns, numbers are standard deviation × 10^−5^.

The concentration of amino acids, carbohydrates and sucrose was significantly (*p* < 0.05) higher in NP than in NF extracts (DF = 3.2, 3.2 and 3.2, respectively; F = 1971010.67, 88610.84 and 61468163.7, respectively) (Fig. 2). No clear distinction (*p* > 0.05) occurred between NP and NF extracts in phenol concentration (DF = 3.2; F = 1139.67). Regarding alkaloids, NF extracts had slightly higher (*p* < 0.05) concentration than NP extracts (DF = 3.2; F = 6085.62).

### Effect of NP-03 fractions on *N. alciformis* pathogenicity to guava seedlings

Among the NP-03 fractions, only NP-03-F2 enabled fungal pathogenicity to guava seedlings (DF = 6; F = 5.4567; *p* < 0.05) (Table 2; Figs. 1D,E). Among the NP-03-F2 fractions, only NP-03-F2-3 and NP-03-F2-4 enabled fungal pathogenicity (DF = 5; F = 3.9741; *p* < 0.05) (Table 3, Fig. 1F).

**Table 2.** Bioassay 2. Effect of fractions of guava root exudates on the pathogenicity of *Neocosmospora falciformis* to guava seedlings. Root exudates were collected from plants parasitized by *Meloidogyne enterolobii*. The methanol-soluble extract NP-03 was submitted to silica column fractionation with the eluents ethyl acetate/methanol (fraction F1), methanol (F2), water (F3), water/acetic acid (F4) and aqueous hydrochloric acid (F5). These fractions were used to prepare the seedlings’ watering solutions.

**Treatments**	**Rootlets’ unrotten, fresh mass**	**Fresh shoot mass**
Uninoculated seedlings watered with sterile, distilled water (control)	64.4^*^a	185.2a
Inoculated seedlings watered with sterile, distilled water (control)	64.7a	198.8a
Inoculated seedlings watered with NP-03-F1	70.1a	159.7a
Inoculated seedlings watered with NP-03-F2	36.2b	87.7b
Inoculated seedlings watered with NP-03-F3	65.7a	184.2a
Inoculated seedlings watered with NP-03-F4	67.2a	189.7a
Inoculated seedlings watered with NP-03-F5	63.5a	198.6a
CV%	15.1	9.7

* Values (all in mg) are the mean of two assays, each with eight replicates (seedlings) per treatment. In the columns, distinct letters indicate significant difference according to the Tukey test at 5%.

**Table 3.** Bioassay 3. Effect of fractions of guava root exudates on the pathogenicity of *Neocosmospora falciformis* to guava seedlings. Root exudates were collected from plants parasitized by *Meloidogyne enterolobii* and submitted to solvent extractions and silica-column fractionation. Column chromatography of NP-03-F2 resulted in fractions -3 through -6, which were used to prepare the seedlings’ watering solutions.

**Treatments**	**Rootlets’ rotten fresh mass**	**Rootlets’ unrotten, fresh mass**	**Fresh shoot mass**
Inoculated seedlings watered with sterile, distilled water (control)	0^*^b	36.3a	141a
Inoculated seedlings watered with NP-03-F2 (control)	12a	13.7b	95.1b
Inoculated seedlings watered with NP-03-F2-3	8.1a	11.4b	93.4b
Inoculated seedlings watered with NP-03-F2-4	12.2a	10.8b	94.6b
Inoculated seedlings watered with NP-03-F2-5	0b	36.4a	157.7a
Inoculated seedlings watered with NP-03-F2-6	0b	36.6a	150.4a
CV%	22.3	13.3	12.6

* Values (all in mg) are the mean of two assays, each with eight replicates (seedlings) per treatment. In the columns, distinct letters indicate significant difference according to the Tukey test at 5%.

### Successive fractionation of NP-03-F2-3+4

**Supplementary Figure 8:**
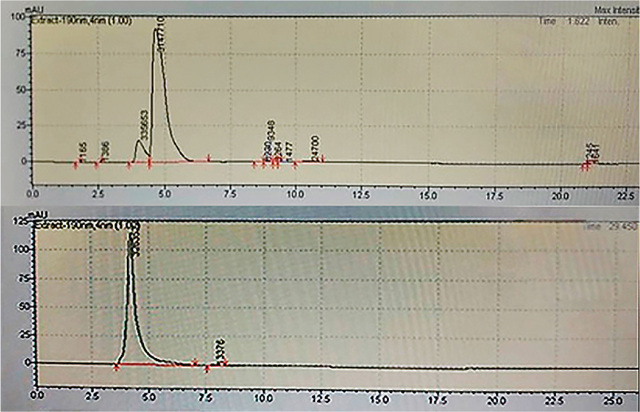
Chromatogram obtained by analyzing the fractions NP-03-F2-3+4-ET-02 (top) and NP-03-F2-3+4-ET-05 (bottom) by high performance liquid chromatography, using a Luna 5-μm column (phenyl-hexyl, 250 by 4.60 mm), with water as the eluent and the UV detector set at 190 nm.

Solvent extractions of NP-03-F2-3+4 resulted in an ethanol-soluble fraction with the greatest mass, which was coded NP-03-F2-3+4-ET (Supplementary Table 3). Fractionation of NP-03-F2-3+4-ET resulted in seven fractions that appeared to be composed of different amounts of two substances. TLC analysis suggested that fractions NP-03-F2-3+4-ET-02 and NP-03-F2-3+4-ET-05 were two pure substances (Supplementary Table 4). HPLC analyses confirmed that fraction NP-03-F2-3+4-ET-05 corresponded to a pure substance, while NP-03-F2-3+4-ET-02 seemed to correspond to another substance that had been contaminated by the substance present in the fraction NP-03-F2-3+4-ET-05 (Supplementary Fig. 8).

### Analyses of the substances present in NP-03-F2-3+4-ET-02 and NP-03-F2-3+4-ET-05

The elemental analysis of NP-03-F2-3+4-ET-05 revealed a C:N ratio of 1.93:5.16, which can be rounded up to 2:5. For NP-03-F2-3+4-ET-02, the ratio was 1.93:8.61, which can be rounded to 2:9. The EDXRF analysis revealed that the substances present in these fractions could only be composed of chemical elements with atomic numbers lower than that of sodium. For these substances, the ^1^H NMR spectra presented broad signals centered at approximately 7.40 ppm. In the ^13^C NMR spectra, a single signal occurred at 159.5 ppm.

An analysis by mass spectrometry of fraction NP-03-F2-3+4-ET-05 showed two main signals in the negative mode, with respective *m/z* (mass/charge) of 147.0171 and 210.0119 u. For NP-03-F2-3+4-ET-02, a signal with *m/z* of 147.0142 u was revealed in the negative mode, while in the positive mode the main signal had *m/z* of 441.0401 u.

### Computational calculations for the structure of 1,5-dinitrobiuret

**Figure 3:**
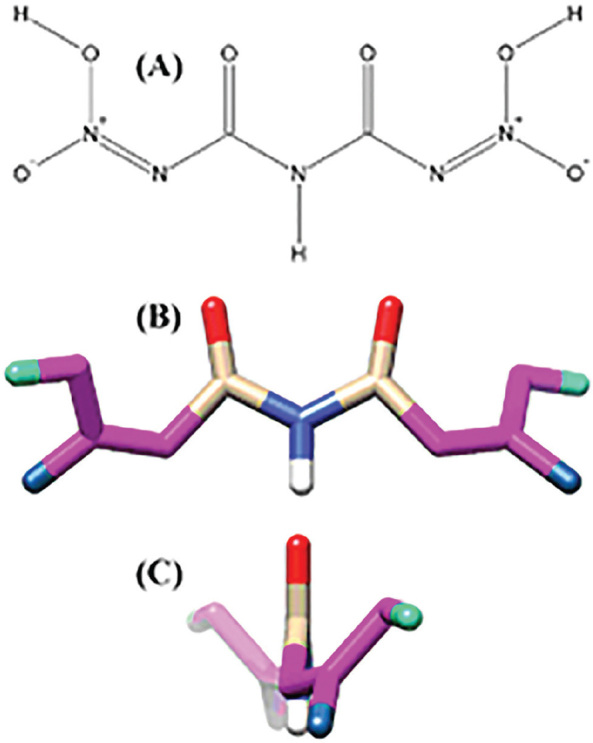
Representation of the most stable conformation of 1,5-dinitrobiuret according to DFT calculations at the theoretical level B3LYP/6-31G(2df,p), implicitly considering the solvent DMSO. A) two-dimensional representation; B) 3D representation with the open chain; C) 3D representation with aligned carbonyls.

Among all the possible conformations and tautomers for 1,5-dinitrobiuret (Supplementary Fig. 1), one corresponded to approximately 89.9% of the total according to the Boltzmann distribution (Fig. 3A). All other structures had values equal to or less than 1%. In the majority structure, there were double bonds between the N atoms of the nitro groups and the neighboring N atoms. A 3D representation revealed a non-planar molecule with a dihedral angle HO-N-N-CO of 11.7 ° (Figs. 3B,C).

### Interaction of 1,5-dinitrobiuret with biuret hydrolase

**Figure 4:**
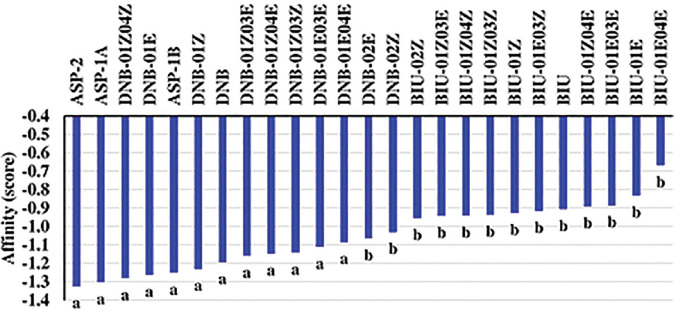
Calculated affinities of 1,5-dinitrobiuret and its tautomers (DNB*); biuret and its tautomers (BIU*); and different protonation states of *N*-formyl-D-aspartic acid (ASP*) for biuret hydrolases.

*N*-formyl-D-aspartic acid, in all protonation states (ASP*), showed equal (*p* < 0.05) affinities to biuret hydrolase, although their values were lower (*p* < 0.05) than those of biuret and its tautomers (BIU*) (Fig. 4). Regarding the calculated values for 1,5-dinitrobiuret and its tautomers (DNB*), most of them were lower (*p* < 0.05) than those calculated for BIU*.

## Discussion

Data from TLC, HPLC, mass spectrometry, NMR, and EDXRF indicated that 1,5-dinitrobiuret was the predominant substance in the fraction NP-03-F2-3+4, which enabled the pathogenicity of *N. falciformis* to guava seedlings. 1,5-dinitrobiuret corresponded to the fraction NP-03-F2-3+4-ET-05.

The EDXRF analysis of NP-03-F2-3+4-ET-05 provided data that was expected for organic substances, although the high N/C ratio observed was unexpected because the overwhelming majority of organic substances of natural origin have a lower N/C ratio than that observed in the present study. Moreover, it is uncommon to obtain only one signal in the ^1^H and ^13^C NMR spectra for organic substances of natural origin. The broad signal at 7.40 ppm in the ^1^H spectrum suggested that H atoms were bonded to heteroatoms only, which necessarily had to be O or N. Regarding the ^13^C spectrum, a single signal at 159.5 ppm suggested a carbonyl group.

**Supplementary Figure 9:**
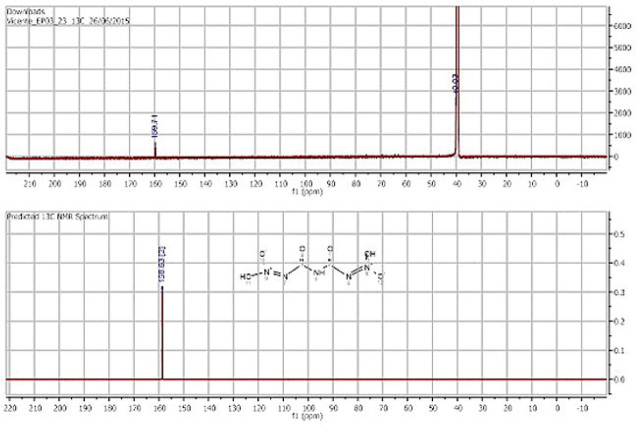
^13^C NMR spectra of 1,5-dinitrobiuret. Top: experimental signal obtained for the fraction NP-03-F2-3+4-ET-05; Bottom: signal obtained from simulation with the software MestReNova 6.0.2.

Searches were made at https://www.stolaf.edu/depts/chemistry/courses/toolkits/380/js/masscalc/ for molecular formulas that could explain the mass spectrum, the high N/C ratio, and the NMR signals found in NP-03-F2-3+4-ET-05. A single formula was obtained, corresponding to dinitrobiuret, which has a monoisotopic mass of 193.008333 u. Therefore, the signal at *m/z* 210.0119 u in the mass spectrum corresponds to [M + OH]^−^, whose calculated monoisotopic mass is 210.011621 u. This differs by only 1 ppm from the experimental value obtained for NP-03-F2-3+4-ET-05. Moreover, the calculated mass for [M – NO_2_]^−^ is 147.015978 u, which differs by only 7 ppm from the *m/z* 147.0171 u that was experimentally obtained. In addition to mass spectrometry, NMR simulation of the ^13^C NMR spectrum of 1,5-dinitrobiuret using the software MNova 6.0.2 (MestreLab Research, https://mestrelab.com) resulted in a single signal at 158.63 ppm for the carbonyl group (Supplementary Fig. 9). This is close to the experimental signal obtained for NP-03-F2-3+4-ET-05. All the experimental data thus support the identification of the substance in the fraction NP-03-F2-3+4-ET-05 as 1,5-dinitrobiuret.

The most stable conformation of 1,5-dinitrobiuret obtained from our DFT calculations is distinct from the pseudo-*trans* conformation proposed by Suntsova et al. (2013). However, these authors performed their calculations in the gas phase, without considering the effect of a solvent in a liquid medium. In our study, the strong polarity of DMSO induced a non-pseudo-*trans* conformation of 1,5-dinitrobiuret to maximize its dipole. This indicates that the two carbon atoms in 1,5-dinitrobiuret must be chemically equivalent, corroborating the single chemical shift observed in the ^13^C NMR spectrum of NP-03-F2-3+4-ET-05.

Biuret has long been known as a contaminant of urea (Jones, 1954; Sanford et al., 1954), and it can be biosynthesized, e.g., from cyanuric acid (Aukema et al., 2020). Microorganisms produce over 200 natural nitro-substituted organic compounds other than biuret (Parry et al., 2011). For instance, *Streptomyces eurocidicus* Witt and Stackebrandt produces 2-nitroimidazole; *S. noursei* Brown, Hazen and Mason produces *N*-nitroglycine; and four bacterial enzymes are involved in the production of nitrated butane-1,2,4-triol (Miyazaki et al., 1968; Niu et al., 2003; Katritzky et al., 2005). In our study, the enrichment of NP root exudates in amino acids, carbohydrates, and sucrose probably favored microorganisms capable of converting urea or other nitrogenous compounds into 1,5-dinitrobiuret. This hypothesis is plausible, since several plant-parasitic nematodes are known to cause changes in root exudates and shifts in the rhizosphere’s microbial community (Wang and Bergeson, 1974; Wang et al., 1975; van Gundy et al., 1977; Yeates et al., 1998, 1999; Denton et al. 1999; Tu et al., 2003; Dromph et al., 2006; Hasse et al., 2007; Wurst et al. 2009; Tian et al., 2015; Lamelas et al., 2020; Xiaolong et al., 2022).

Some soil bacteria use the enzyme biuret hydrolase to decompose biuret (Cameron et al., 2011; Aukema et al., 2020). Interestingly, transgenic rice plants overexpressing biuret hydrolase have a higher tolerance to biuret toxicity (Ochiai et al., 2020), suggesting that biuret hydrolase is important in circumventing the phytotoxicity of biuret. In the present work, *in silico* analysis showed that 1,5-dinitrobiuret complexes with biuret hydrolase have an energy equivalent to *N*-formyl-D-aspartic acid, which is the enzyme’s inhibitor. Hence, we hypothesize that 1,5-dinitrobiuret inhibits biuret hydrolase, leading to a biuret phytotoxicity that hampers guava root physiology and immunity to *N. falciformis*. It is worth mentioning that 1,5-dinitrobiuret may not be the only substance relevant in the synergistic interaction of *M. enterolobii* and *N. falciformis,* since the fraction NP-04 was not characterized.

In addition to 1,5-dinitrobiuret, the fraction NP-03-F2-3+4 had another substance, corresponding to the fraction NP-03-F2-3+4-ET-02. In this fraction, a single signal was observed in the mass spectrometry analysis in the negative mode, with m/z of 147.0142 u. This is compatible with nitrobiuret minus H^+^. However, in the positive mode, the main signal had m/z of 441.0401 u, which seems incompatible with the structure of nitrobiuret. Furthermore, the structure of nitrobiuret does not explain the C:N ratio observed in the elemental analysis of approximately 2:9. Hence, the substance in the fraction NP-03-F2-3+4-ET-02 remains unidentified.

In addition to a putative shift in the guava rhizobiome, the enrichment of NP root exudates in amino acids, carbohydrates, and sucrose may contribute to satisfying *N. falciformis’*s nutritional needs. *In vitro*, NP root exudates favored *N. falciformis*’s mycelial growth and propagule production (Gomes et al., 2013). Nonetheless, it is not clear whether the enriched exudates benefit the fungus directly under natural conditions, since root exudates are avidly disputed by the vast array of microorganisms that constitute the rhizobiome. With regard to alkaloids, NP extracts had a slightly lower concentration than NF extracts. No clear distinction occurred with regard to phenols between NP and NF extracts.

In conclusion, this study offers an in-depth characterization of changes induced by *M. enterolobii* in guava root exudates, in association with root rotting caused by *N. falciformis*. Parasitism by *M. enterolobii* leads to guava root exudates enriched in amino acids, carbohydrates, and sucrose, a condition that likely favors microorganisms capable of producing 1,5-dinitrobiuret in the rhizosphere. The accumulation of biuret in the rhizosphere may hamper guava root physiology and immunity to *N. falciformis*. At this point, the microorganisms and mechanisms involved in guava decline are not entirely known, but our results underscore the contemporary view that plant soilborne diseases should be viewed as an outcome of interactions between a plant, its root exudates and the rhizobiome.

## Figures and Tables

**Supplementary Table 1.** Dry mass and relative proportion of extracts obtained from guava (*Psidium guajava*) root exudates after extraction with organic solvents and water. The plants, cultivated in a growth chamber, were either parasitized by *Meloidogyne enterolobii* (coded NP) or nematode-free (NF)

**Extract code**	**Solvent used**	**Dry mass (g)**	**Relative mass (%)^a^**
NP-01	Hexane	0.01	0.04
NF-01		0.018	0.13
NP-02	Ethyl acetate	0.145	0.69
NF-02		0.086	0.6
NP-03	Methanol	12.72	60.42
NF-03		7.443	51.8
NP-04	Water	8.177	38.84
NF-04		6.824	47.5

a In relation to the total NP or NF dry mass used for solvent extraction.

**Supplementary Table 2.** Dry mass and relative proportion of fractions obtained from chromatographic fractionation of NP-03-F2 with different eluents. NP-03-F2 is a fraction of the methanol-soluble extract of root exudates of guava (*Psidium guajava*) plants cultivated in a growth chamber and parasitized by *Meloidogyne enterolobii*.

**Fraction code**	**Eluent**	**Column fractions**	**Dry mass (g)**	**Relative mass (%)^a^**
NP-03-F2-1	Ethyl acetate/methanol (7:3)	1–4	0.0004	0.03
NP-03-F2-2	Ethyl acetate/methanol (7:3)	5 and 6	0.051	3.5
NP-03-F2-3	Ethyl acetate/methanol (7:3)	7–9	0.448	30.73
NP-03-F2-4	Ethyl acetate/methanol (7:3)	10–14	0.507	34.77
NP-03-F2-5	Ethyl acetate/methanol (7:3)	15–19	0.115	7.89
NP-03-F2-6	Ethyl acetate/methanol (3:7)	20–23	0.323	22.15
NP-03-F2-7	Ethyl acetate/methanol (3:7)	24–29	0.006	0.41
NP-03-F2-8	Water	single	0.008	0.55

a Relative to the sum of all fractions’ mass.

**Supplementary Table 3.** Dry mass and relative proportion of fractions obtained from extraction of NP-03-F2-3+4 with different solvents. NP-03-F2-3+4 is a chromatographic fraction of root exudates of guava (*Psidium guajava*) plants cultivated in a growth chamber and parasitized by *Meloidogyne enterolobii*.

**Fraction code**	**Solvent used**	**Dry mass (mg)**	**Relative mass (%)^a^**
NP-03-F2-3+4-DI	Diethyl ether	4.9	1.32
NP-03-F2-3+4-ET	Ethanol	291.6	78.42
NP-03-F2-3+4-ME	Methanol	72	19.37
NP-03-F2-3+4-IN	Insoluble	3.3	0.89

a In relation to the mass of the fraction NP-03-F2-3+4.

**Supplementary Table 4.** Dry mass and relative proportion of fractions obtained from chromatographic separation of NP-03-F2-3+4-ET. NP-03-F2-3+4-ET is an ethanol-soluble fraction of root exudates of guava (*Psidium guajava*) plants cultivated in a growth chamber, and parasitized by *Meloidogyne enterolobii*.

**Fraction code**	**Column fractions**	**Dry mass (mg)**	**Relative mass (%)^a^**
NP-03-F2-3+4-ET-01	1–10	40.7	13.6
NP-03-F2-3+4-ET-02	11–14	36.2	12.1
NP-03-F2-3+4-ET-03	15–20	45.1	15
NP-03-F2-3+4-ET-04	21–25	22.6	7.5
NP-03-F2-3+4-ET-05	26–32	89.4	29.8
NP-03-F2-3+4-ET-06	33–50	16.6	5.5
NP-03-F2-3+4-ET-07	51 (methanol)	45.8	15.3

a In relation to the mass of NP-03-F2-3+4-ET.

## Supplementary Material

### Quantification of free amino acids, soluble carbohydrates, sucrose, phenols and alkaloids in the methanol and water NP and NF extracts

Quantification of amino acids, soluble carbohydrates, and sucrose (mg/ml) was performed using the method of Passos (1996) modified by Campos et al. (2012). Aliquots (100 mg) of the extracts were subjected to three consecutive extractions with 13 ml of a solution of methanol/chloroform/water 12:5:3 (v/v/v). For each extract, the three suspensions were pooled, filtered through a cotton wool plug, added to a mixture of chloroform (30 ml) and water (60 ml), and vigorously stirred. After standing for a few minutes, the aqueous phase was separated by decantation, concentrated to dryness on a rotary evaporator, and lyophilized. For each extract, the residues obtained were dissolved in 25 ml of water, hereafter referred to as solution A.

To quantify amino acids in the extracts, three 0.5-ml aliquots were taken from the respective solution A. These were transferred to glass test tubes containing 1 ml of a previously prepared ninhydrin solution (25 ml NaOAc.3H_2_O at 1.36 g/ml (pH 5.5); 20 ml of acetic acid; 75 ml of ethylene glycol monomethyl ether; 0.2 g of ninhydrin; 30 mg hydrindantin). After shaking, the test tubes were heated in a water bath at 100 °C for 15 min. The test tubes were cooled to room temperature, and 3 ml of a 50% aqueous ethanol solution was added to each tube. The absorbance was measured at 570 nm in an SP1105^®^ spectrophotometer (Spectrum, China) using glass cuvettes with a 1-cm path length. The value was converted into amino acid concentration by comparison with a standard curve based on L-leucine.

To quantify soluble carbohydrates, three aliquots (1 ml) of each solution A were transferred to glass test tubes, to which were added 0.5 ml of an aqueous (0.05 g/ml) phenol solution and 2.5 ml of concentrated sulfuric acid. After 10 min, the tubes were heated in a water bath at 30 °C for 15 min. The absorbance was measured at 490 nm, and the value was converted to carbohydrate concentration by comparison with a standard curve based on D-glucose.

To quantify sucrose, three 1-ml aliquots of each solution A were transferred to glass test tubes and lyophilized. Sterile distilled water (0.1 ml) and 0.1 ml of aqueous 30% (g/ml) potassium hydroxide were added to the tubes, which were heated in a water bath at 100 °C for 10 min and cooled to room temperature. Five ml of anthrone solution (600 mg of anhydrous anthrone; 270 ml of concentrated sulfuric acid; 30 ml of distilled water) was added to each tube. The tubes were kept in a water bath at 40 °C for 10–15 min, and the absorbance was measured at 620 nm. The value was converted to sucrose concentration by comparison with a standard curve based on sucrose.

Phenol quantification (mg/ml) was performed by the method of the Association of Official Analytical Chemists (Anonymous, 1996), modified. One hundred mg of NP-03, NP-04, NF-03, and NF-04 extracts were separately dissolved in 12.5 ml of a 1:1 (v/v) aqueous methanol solution in a glass flask. A condenser was connected to the flask, which was heated to reflux for 15 min. The solution was filtered through a cotton wool plug, and the solid residue underwent the same process twice. The extracts were pooled and diluted in water to a final volume of 40 ml. Aliquots (20 μl) were placed in three glass test tubes, to which were added 1.68 ml of distilled water, 200 μl of aqueous 0.02 g/ml sodium carbonate solution, and 100 μl of Folin-Denis reagent (2.5 g of dihydrated sodium tungstate; 0.5 g of phosphomolybdic acid; 1.25 ml of concentrated phosphoric acid; and the necessary amount of water to reach a final volume of 25 ml). The absorbance was measured at 720 nm, and the value was converted to phenol concentration by comparison to a standard curve based on tannic acid.

Alkaloids were quantified (mg/ml) by the method of Sreevidya and Mehrotra (2003). Three hundred mg of NP-03, NP-04, NF-03, and NF-04 extracts underwent four extractions (10 min each) with 10 ml of a water/acetic acid solution 98:2 (v/v). From the pooled liquid phases, 5-ml aliquots were added to glass test tubes with 2 ml of Dragendorff reagent (40 ml of Bi(NO_3_)_3_ at 0.02 g/ml; 30 ml of acetic acid-water in a 1:2 (v/v) proportion; 8 g of potassium iodate). The resulting mixture was centrifuged at 818 *g*, and the precipitate was washed twice with 4 ml of ethanol. Two ml of a 1% (g/ml) aqueous solution of sodium sulfide was added to the precipitate. The mixture underwent centrifugation (818 g), and the precipitate was dissolved in 2 ml of concentrated nitric acid. The resulting solution was diluted with distilled water up to 10 ml, from which three 1-ml aliquots were withdrawn and added to 5 ml of a 3% (g/ml) aqueous solution of thiourea. The absorbance of this solution was measured at 435 nm, and the value was converted to alkaloid concentration by comparison to a standard curve based on brucine.

### Computational calculations applied to 1,5-dinitrobiuret

The chemical structure of the 1,5-dinitrobiuret tautomers was drawn with the Chemsketch 12.01 program (Advanced Chemistry Development, Toronto, Canada) and saved in mol format for later conversion to the sdf format using the computer program OpenBabel 2.3.2 (O’Boyle et al., 2011).

From the sdf format, conformational searches were performed using the Open3Dalign 2.282 program (Tosco et al., 2011). One thousand molecular dynamic simulations were performed using the Merck Molecular Force Field 94^®^ (MMFF94) with temperature set at 1,000 ºC and 1,000 steps of 1 fs in each simulation. Solvent (DMSO) was implicitly considered. The most stable conformation of each chemical structure, as well as those up to 10 kcal/mol away from the most stable, were optimized using the mopac 2016 program (http://openmopac.net/, J.J.P. Stewart, Stewart Computational Chemistry, Colorado Springs, CO, USA), employing Hamiltonian PM7. DMSO was implicitly considered through the conductor-like screening model (COSMO) (Klamt and Shüürmann, 1993). The total energy of the optimized structures was used to calculate the Boltzmann distributions. The structure that corresponded to approximately 89.9% of the total structures was subjected to optimization with the NWChem 6.8.1 program (Aprà et al., 2020) by DFT calculation at the theoretical level B3LYP/6-31G (2df,p). The solvent was implicitly considered using COSMO.

### Interaction of 1,5-dinitrobiuret biuret hydrolase

1,5-dinitrobiuret and its tautomers, biuret and its tautomers, and different protonation states of *N*-formyl-D-aspartic acid were submitted to a conformational search. However, instead of DMSO, water was used as the solvent. The three-dimensional (3D) structures (in pdb format) of biuret hydrolases 5bk6, 6azq, and 6azs (Esquirol et al., 2018) were obtained from the RCSB Protein Data Bank (https://www.rcsb.org/) to be aligned with the computer program Lovoalign 21.027 (Martínez et al., 2007). The most stable conformations of 1,5-dinitrobiuret and its tautomers and biuret and its tautomers were aligned to biuret that had been experimentally complexed to biuret hydrolase 6azq. The most stable conformations of the different protonation states of *N*-formyl-D-aspartic acid were aligned to the molecules of this acid that had been experimentally complexed to biuret hydrolase 6azs. These alignments were performed using UCSF Chimera 1.15 (Pettersen et al., 2004). Examples of these alignments can be seen in Supplementary Figures 5–7.

The 3D structures of biuret hydrolases 5bk6, 6azq, and 6azs, without any ligand, ion, or solvent, were combined with the conformation of chemical structures aligned to biuret or *N*-formyl-D-aspartic acid to form various complexes. These complexes were submitted to an optimization step with Chimera 1.15 by adding charges to the protein atoms according to the Amber 14SB force field. The Antechamber program (Wang et al., 2006), inserted in Chimera 1.15, was used to add charges to other atoms. Semi-empirical calculations (AM1) were performed with bond charge correction and parameterized to reproduce ab initio (HF/6-31G*) electrostatic potentials. Other parameters for non-protein atoms were obtained from the General Amber Force Field (GAFF) (Wang et al., 2004) shipped with Chimera 1.15. The optimization of each complex was done with 300 steepest descent steps, followed by 30 conjugated gradient steps. Step sizes equal to 0.02 Å and updates at intervals of 10 steps were used. Cyscore 2.0.3 (Cao and Li, 2014) was used to calculate the affinities of the substances to the enzymes.
